# Measuring Distribution Similarities Between Samples: A Distribution-Free Overlapping Index

**DOI:** 10.3389/fpsyg.2019.01089

**Published:** 2019-05-21

**Authors:** Massimiliano Pastore, Antonio Calcagnì

**Affiliations:** Department of Developmental and Social Psychology, University of Padova, Padova, Italy

**Keywords:** overlapping, distribution free, empirical distributions, effect size, R-package

## Abstract

Every day cognitive and experimental researchers attempt to find evidence in support of their hypotheses in terms of statistical differences or similarities among groups. The most typical cases involve quantifying the difference of two samples in terms of their mean values using the *t* statistic or other measures, such as Cohen's *d* or *U* metrics. In both cases the aim is to quantify how large such differences have to be in order to be classified as notable effects. These issues are particularly relevant when dealing with experimental and applied psychological research. However, most of these standard measures require some distributional assumptions to be correctly used, such as symmetry, unimodality, and well-established parametric forms. Although these assumptions guarantee that asymptotic properties for inference are satisfied, they can often limit the validity and interpretability of results. In this article we illustrate the use of a distribution-free overlapping measure as an alternative way to quantify sample differences and assess research hypotheses expressed in terms of Bayesian evidence. The main features and potentials of the overlapping index are illustrated by means of three empirical applications. Results suggest that using this index can considerably improve the interpretability of data analysis results in psychological research, as well as the reliability of conclusions that researchers can draw from their studies.

## 1. Introduction

Overlapping can be defined as the area intersected by two or more probability density functions and offers a simple way to quantify the similarity (or difference) among samples or populations which are described in terms of distributions. Intuitively, two populations (or samples) are similar when their distribution functions overlap. The simplicity of the overlapping concept makes the use of this index particularly suitable for many applications such as, for example, the comparison of probability distributions by exploring the amount of common area shared on the same domain. In addition, overlapping can serve as a measure to estimate distances among clusters/networks of data, or alternatively to measure similarities among datasets (e.g., Goldberg et al., 2010).

Since the first statistical contributions which introduce the idea of overlapping by Gini and Livada (1943) and Weitzman (1970), the overlapping index has been applied to several research problems involving, for instance, data fusion (Moravec, 1988), information processing (Viola and Wells, 1997), applied statistics (Inman and Bradley, 1989), and economics (Milanovic and Yitzhaki, 2001). The idea of overlapping has also been independently used in psychology, especially in the definition of some effect size measures. In this regard, overlapping has served as a basis for Cohen's *U* index (Cohen, 1988), McGraw and Wong's *CL* measure (McGraw and Wong, 1992), and Huberty's *I* degree of non-overlap index (Huberty and Lowman, 2000). Albeit compelling, these indices require some distributional assumptions to be properly met (e.g., symmetry of the distributions, unimodality, same parametric family). While these assumptions guarantee that asymptotic properties for inference are satisfied, they can somewhat limit the application of overlapping-based metrics.

In the current paper, we will describe how overlapping can be constructed in more general terms without resorting to the use of strong distributional assumptions (see Inman and Bradley, 1989), thus allowing us to adopt overlapping in many real-world applications. Specifically, our aim is two-fold. First, we introduce a distribution-free overlapping index which can be used in many data analysis applications, especially those where researchers need to quantify the magnitude of some phenomena like differences, distances, and evidence. Second, we present an R-based software implementation of the distribution-free overlapping which is index, simple and flexible enough to be used in statistical analysis of psychological data. Moreover, we will show how the distribution-free overlapping index can also be used in a Bayesian perspective, particularly in posterior data analysis (e.g., when researchers need to quantify evidence in favor of a particular hypothesis, to assess the leveraging of prior on posterior conditioned to a given data sample).

The remainder of this article is structured as follows. First, we briefly review some statistical definitions and properties of the overlapping index and shed light on relevant relations with other effect size measures. Next, we illustrate our implementation of the overlapping measure, developed for the open source statistical software R (R Core Team, 2018). Afterwards, we motivate the need for a distribution-free overlapping index by discussing three general examples which are applicable to psychological research studies. Finally, we conclude the article by summarizing potentials and limitations of our proposal.

## 2. The overlapping index

In this section, we shortly provide statistical definitions and properties of the overlapping index. To begin with, let us assume two real probability density functions *f*_*A*_(*x*) and *f*_*B*_(*x*). The overlapping index η : ℝ^*n*^ × ℝ^*n*^ → [0, 1] is defined as follows:

(1) $$\begin{array}{r} \eta \left(A,B\right)=\int_{{\mathbb{R}}^{n}}\min\left[f_{A}\left(x\right),f_{B}\left(x\right)\right]dx \end{array}$$

where the integral can be replaced by summation in the discrete case. As for any measure of association, η(*A, B*) is normalized to one, with η(*A, B*) = 0 indicating that *f*_*A*_(*x*) and *f*_*B*_(*x*) are distinct (i.e., the support of the distributions of *A* and *B* does not present interior points in common). In this sense, η(*A, B*) provides a way to quantify the agreement between *A* and *B* in terms of their density functions (Inman and Bradley, 1989). The index η(*A, B*) is proven to be invariant under strictly increasing and differentiable transformation on the supports of *A* and *B* (Schmid and Schmidt, 2006). Moreover, the following alternative definition sheds light on how overlapping can work as a dissimilarity measure:

(2) $$\begin{array}{l} \eta (A,B)=1-\delta (A,B)\\ =1-\left(\frac{1}{2}\int_{{\mathbb{R}}^{n}}|f_{A}(x)-f_{B}(x)|dx\right) \end{array}$$

with | . | indicating the absolute value operator. Interestingly, Equation 2 links the overlapping index to the well-known Kullback-Leibler divergence (Kullback and Leibler, 1951) and Bhattacharyya's distance (Bhattacharyya, 1943). However, while the latter metrics require some distributional assumptions to be correctly applied (e.g., symmetry and uni-modality of the density functions), the overlapping index, η(*A, B*), does not strictly require distributional assumptions about *f*_*A*_(*x*) and *f*_*B*_(*x*) (Inman and Bradley, 1989; Clemons and Bradley, 2000). This makes η(*A, B*) flexible enough to be applied in many practical situations. Additionally, since overlapping is defined on probability density function, in some circumstances η(*A, B*) can be interpreted as a misclassification index (Schmid and Schmidt, 2006), as shown by the following results:

(3) $$\begin{array}{l} \eta (A,B)\;=\int_{{\mathbb{R}}^{n}}\min[f_{A}(x),f_{B}(x)]\;dx\\ =\int_{{\mathbb{R}}^{n}}f_{A}(x)\cdot I_{f_{A}(x)<f_{B}(x)}\;dx+\\ +\int_{{\mathbb{R}}^{n}}f_{B}(x)\cdot I_{f_{B}(x)\leq f_{A}(x)}\;dx \end{array}$$

with *I*(.) being an indicator function. In this case, the term *f*_*A*_(*x*) < *f*_*B*_(*x*) represents the case of choosing *f*_*B*_ when *f*_*A*_ is the true density, *f*_*B*_(*x*) ≤ *f*_*A*_(*x*) indicates the complementary case, whereas the integrals over the indicator functions *I*(.) provide an estimate of error probabilities of the classification. Note that this definition makes η close to the Vargha and Delaney's effect size (Vargha and Delaney, 2000; Peng and Chen, 2014). The overlapping index η(*A, B*) can be computed either analytically, when the densities *f*_*A*_(*x*) and *f*_*B*_(*x*) are known, or approximately, when researchers have no particular knowledge about the parametric form of *f*_*A*_(*x*) and *f*_*B*_(*x*). In the particular case where *f*_*A*_ and *f*_*B*_ are Normal under the constraint $\sigma_{A}^{2}=\sigma_{B}^{2}=\sigma^{2}$, the index η(*A, B*) becomes proportional to Cohen's *d* (Inman and Bradley, 1989), as follows:

(4) $$\begin{array}{l} \eta (A,B)\;=\int_{{\mathbb{R}}^{n}}\min[N(x|\mu_{A},\sigma^{2}),N(x|\mu_{B},\sigma^{2})]\;dx\\ =2\Phi \left(-\frac{\left|\mu_{A}-\mu_{B}\right|}{2\sigma }\right) \end{array}$$

where N means the Normal probability law, μ_*A*_ and μ_*B*_ indicate the location parameters, σ^2^ is the pooled variance whereas Φ(.) is the standard Normal distribution function. It is worthwhile to note that the argument |μ_*A*_ − μ_*B*_|/2σ is the standardized mean difference effect size. Although compelling, these results cannot be derived when sample data do not suggest any reasonable parametric form for *f*_*A*_ and *f*_*B*_. In all these cases, approximations need to be introduced in order to estimate the unknown densities and compute the integrals in the η(*A, B*) formula. In the next paragraph, we will describe a simple distribution-free approximation to compute η(*A, B*), which can be adopted in many practical cases of data analysis.

### 2.1. Distribution-Free Approximation of η

Let **x** = (*x*_1_, …, *x*_*i*_, …, *x*_*n*_) and **y** = (*y*_1_, …, *y*_*i*_, …, *y*_*n*_) be realizations of *X* and *Y*. Then the unknown densities *f*_*X*_ and *f*_*Y*_ can be estimated via Kernel density estimators:

(5) $${\hat{f}}_{X}(x)=n^{-1}\sum_{i=1}^{n}K\left(\frac{x-x_{i}}{\beta }\right)$$

(6) $${\hat{f}}_{Y}(y)=n^{-1}\sum_{i=1}^{n}K\left(\frac{y-y_{i}}{\beta }\right)$$

where K is the Kernel function (e.g., gaussian, epanechnikov, biweight) and β is the usual bandwidth parameter. Substituting Equations 5-6 into Equation 1 yields the following approximation for the overlapping index:

(7) $$\begin{array}{r} \hat{\eta }\left(X,Y\right)=\int_{{\mathbb{R}}^{n}}\min\left[{\hat{f}}_{X}\left(z\right),{\hat{f}}_{Y}\left(z\right)\right]dz \end{array}$$

where the integral $\int_{\mathbb{R}}f\left(z\right)dz$ can be computed numerically (e.g., trapezoidal rule, numerical quadrature) or using the average operator on a discretization of the integration support. The approximation in Equation 7 depends upon the choice of K and β. For instance, Clemons and Bradley (2000) have shown that the Normal Kernel with the normal reference rule for the bandwidth computation (Silverman, 1986) tends to work adequately with limited bias. In a similar way, Schmid and Schmidt (2006) investigated the behavior of $\hat{\eta }$ in a more extended simulation study, assessing bias and standard deviations as a result of choosing K and β. In a more complicated scenario, like for the multivariate case, the choice of K and β can be performed via sensitivity analysis.

**Figure 1:**
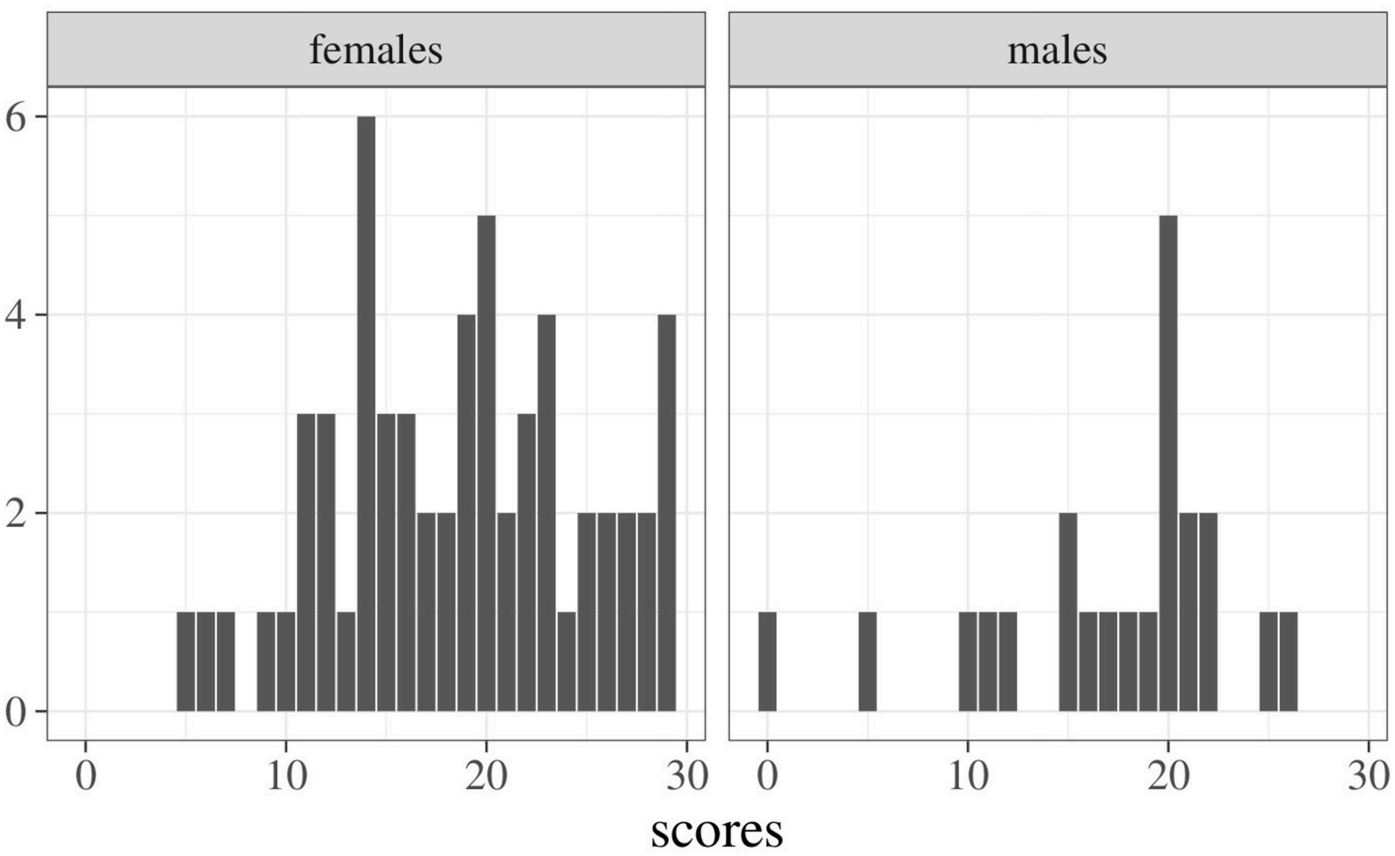
Example: Frequency distributions of exam scores for males and females.

**Figure 2:**
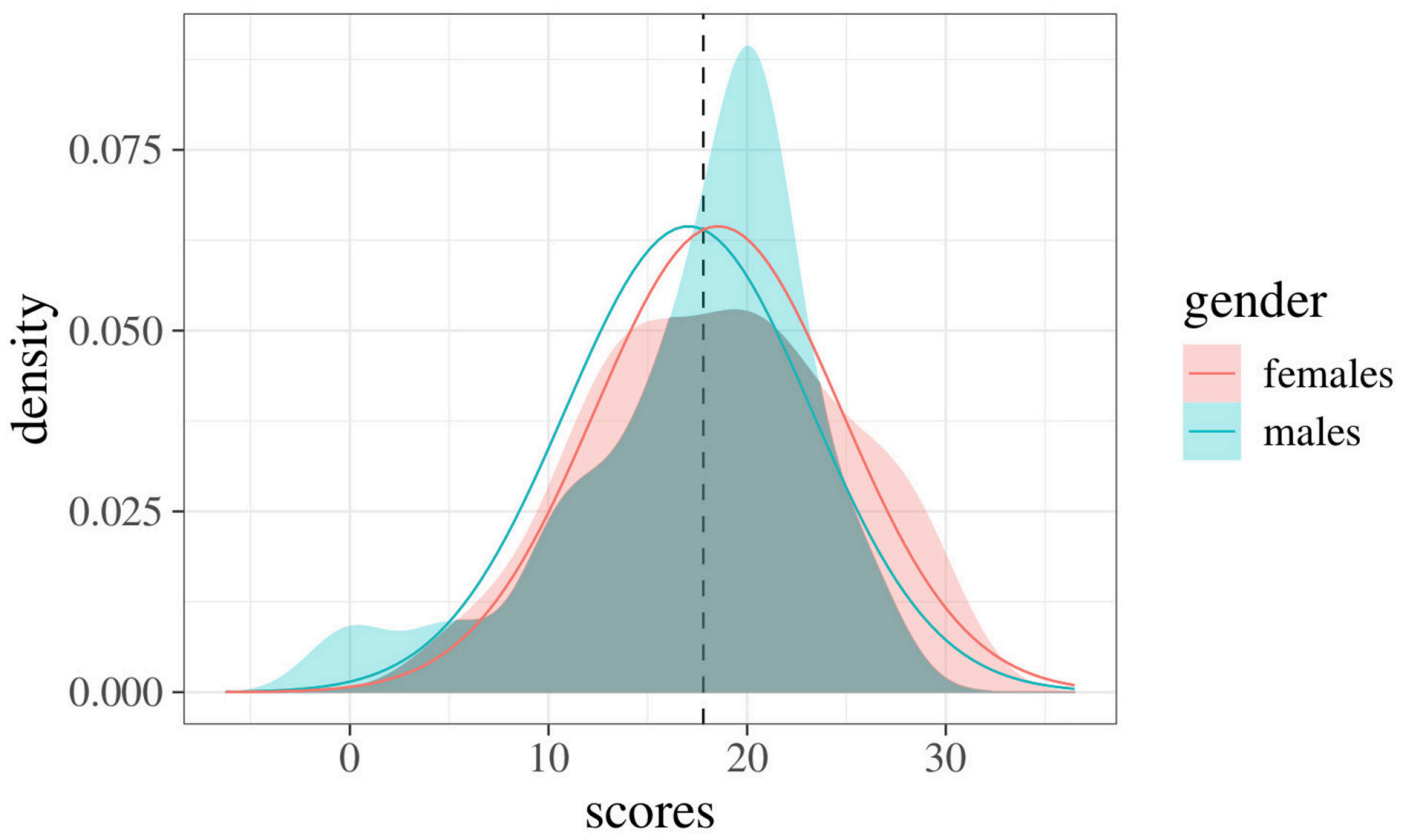
Empirical densities for both male and female groups. Continuous curves represent the true expected densities under Cohen's *d* metrics, whereas the dotted line indicates the intersection point between these expected densities.

To better appreciate the potentials of the index in Equation 7, consider the following example regarding students' scores on a given exam (*N* = 81, 22 males and 59 females). Figure 1 shows the frequency distributions of scores for females (left panel) and males (right panel). A common way to evaluate differences across samples is to use a test for mean differences (i.e., *t*-test) which, in this case, yields the following results: ${\bar{x}}_{\text{m}}=17.05$ (s.d. 6.28), ${\bar{x}}_{\text{f}}=18.54$ (s.d. 6.27), *t*_(79)_ = 0.96, *p* = 0.34. An estimate of effect size is performed by Cohen's *d*, obtaining a small effect: *d* = −0.24, 95% CI [−0.73, 0.25] (Cohen, 1988). Figure 2 shows the empirical densities estimated via the Gaussian Kernel method (shaded colored areas) against the expected densities based on the normality distribution assumption (solid colored curves). Interestingly, the overlapping index computed via Equation 4 is about η = 0.91 even though the empirical densities show irregular overlaps (i.e., the density for the male group is more right-skewed then the density for the female group). Indeed, by using the approximation index in Equation 7 we get $\hat{\eta }=0.66$, which is lower when compared to the expected one. This is of particular relevance, for instance, when working with small and non-Gaussian samples—like those in the current example—where the true overlapping index tends to be biased upward. The sample properties of the overlap estimator have been extensively investigated by Schmid and Schmidt (2006), who proved its properties under mild regularity conditions. The reader can refer to these authors for further and formal details.

## Computing the Distribution-Free $\hat{\eta }$ Index

The overlapping index in Equation 7 can be easily computed using the freely-available R-package overlapping (Pastore, 2018), which is downloadable from CRAN (https://cran.r-project.org/package=overlapping) and includes other utilities for statistical computing and graphical representations as well (note that graphics are performed by means of the R-package ggplot2; Wickham, 2009). We will now shortly describe how the overlapping package can be used to estimate the overlapping index.

The main function of the package is overlap(), and it requires as input a list of at least two elements containing the observed data and, optionally, the number of equally spaced points (or bins) for the integral computations (default is 1,024). Let us consider the following example code for illustrative purposes: 
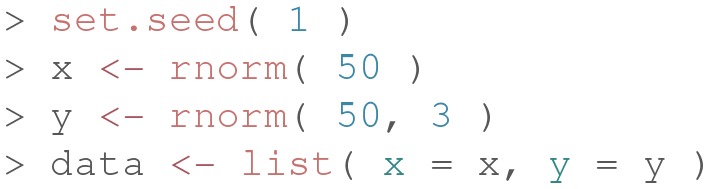
 First, we simulate empirical data by randomly sampling 50 values from a standardized normal distribution (i.e., vector x) and 50 values from a normal distribution with mean 3 and variance 1 (i.e., vector y). Next, we create a list including these two data vectors.

Finally, we compute the overlapping index as follows: 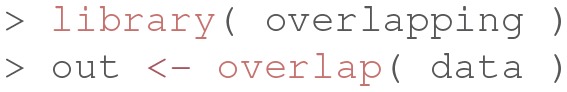 The command library() loads the package, and the function overlap() estimates the overlapping area between the two distributions in the data-list. The function returns a new object-list, out, which contains three different objects: DD (a data frame with information used for computing overlapping), OV (the estimated overlapping index), xpoints (abscissas of intersection points among the density curves). The objects can be visualized via the command str(out). For further details, see the manual at https://cran.r-project.org/web/packages/overlapping/overlapping.pdf.

At this stage, the object OV contains the estimated overlapping index $\hat{\eta }$: 
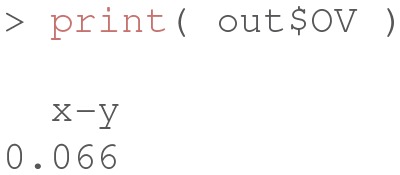
 Finally, the graphical representation of the overlapping area between d1 and d2 can be easily produced via the syntax overlap( …, plot = TRUE ).

The overlap() function works also when more than two vectors of data are available (i.e., more than two sample groups), as shown by the following example, where the overlapping indices are computed pairwise: 
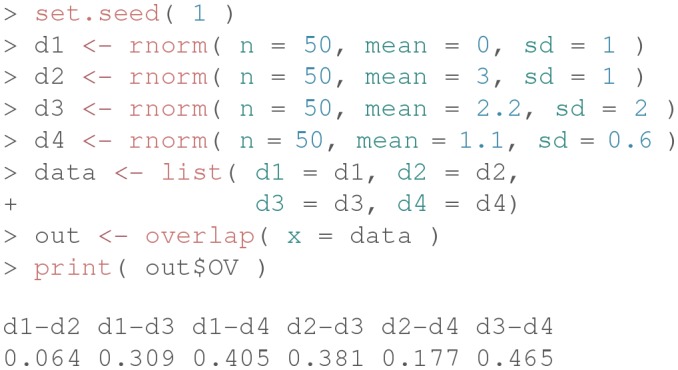
 More technically, the $\hat{\eta }$ index in Equation 7 is computed as follows. Given a set of *K* random vectors, the densities ${\hat{f}}_{1},\ldots ,{\hat{f}}_{K}$ are first computed via Gaussian-Kernel estimation on a finite and real support *z* with *N* equally spaced points [specified via the parameter nbins in the function overlap()]. Next, the indefinite integral in the formula is approximated with a sum of definite integrals on all the subintervals/classes defined on *z*. The definite integrals are finally computed using the well-known trapezoidal rule. Note that the approximation of the indefinite integral, which would otherwise be missed in the case of using more expensive numerical quadrature techniques, is due for the sake of computational simplicity, and it is valid for large values of *N* (as such, we set nbins=1,024 in the overlap() function by default). More details are available by typing ?density in the R console.

## 3. Examples

In this section we describe three illustrative applications of the overlapping index. In particular, in the first one we consider a typical experimental case of two independent groups with non-normally distributed scores and different variances. In the second example, we consider a mediation model in which an indirect effect is hypothesized to differ in two independent groups. Finally, in the third example we present a Bayesian application comparing a (theoretical) prior distribution with a (empirical) posterior distribution.

### 3.1. Example 1: Comparing Experimental Groups With Non-normal Scores

Consider the case of comparing two experimental groups, *x* and *y*, with samples sizes *N*_*x*_ = 35 and *N*_*y*_ = 30. In the *x* group, the mean score is about $\bar{x}=1.57$ with s.d. 1.78, whereas in the *y* group, $\bar{y}=3.44$ with s.d. 4.32. When comparing the two groups with a *t*-test, the result is biased as the groups differ in variance (i.e., in the *y* group the variance is about six times higher than in *x*).

**Figure 3:**
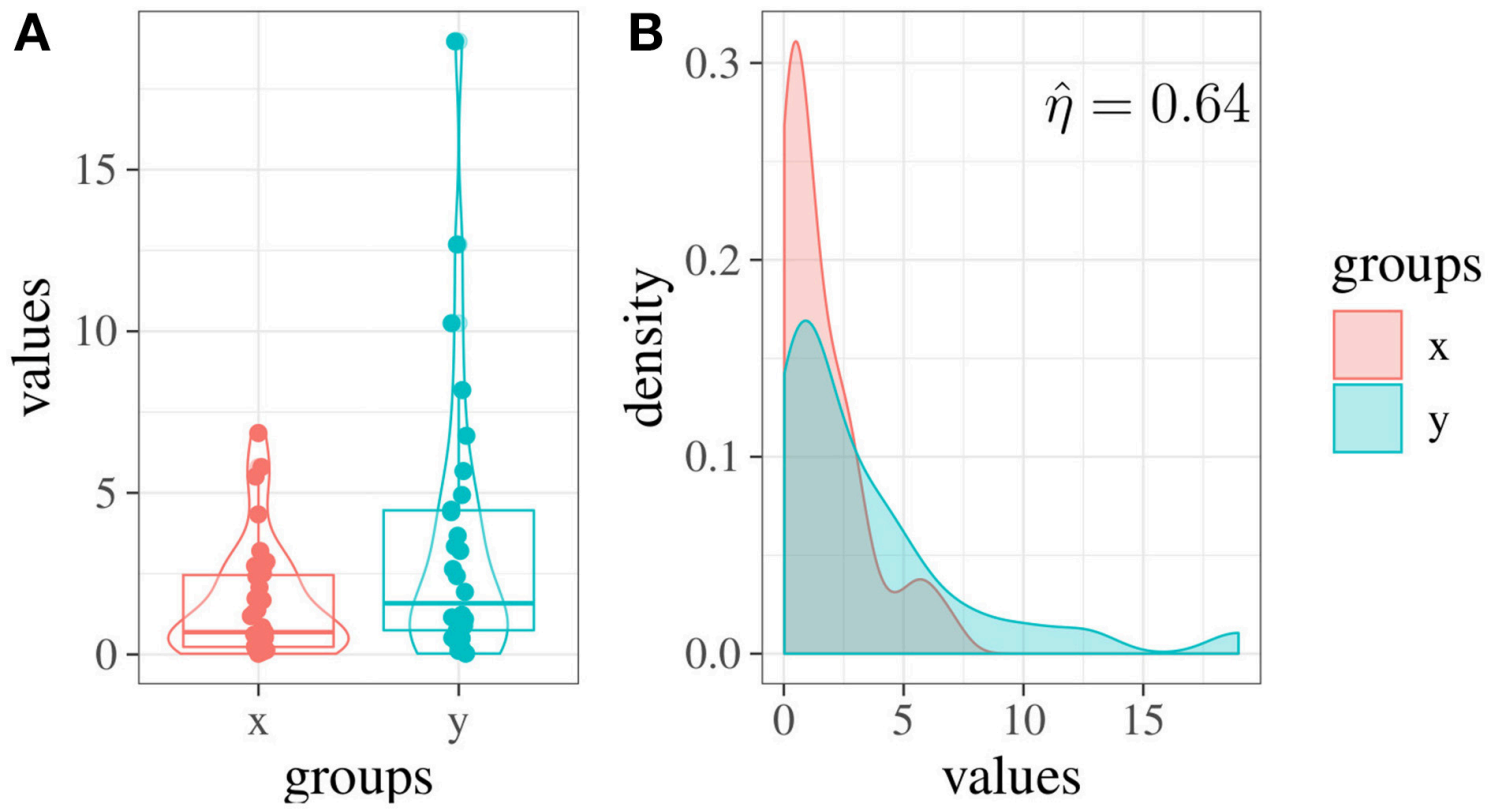
Example 1: **(A)** Score distributions in two simulated experimental groups (*x* and *y*). **(B)** Estimated densities of the two groups and overlapping area.

Figure 3A, shows the pirateplots (Phillips, 2017) of the two groups. We can note that the two distributions differ also in their shape (Figure 3B): in this case, the skewness of *x* is about 1.41, whereas the skewness of *y* is about 1.93.

By performing a Welch two sample *t*-test, we obtain *t*_(37.35)_ = −2.21, *p* = 0.03, leading to a significant result. Cohen's *d* equal to 0.59 indicates a medium effect. However, by computing the overlapping index on this data we obtain $\hat{\eta }=0.64$ (see Figure 3B), which supports the fact that the two groups are less different than they appeared to be. Hence, in this example, the estimation of the overlapping index has reduced the risk of obtaining biased results due to the presence of outliers.

### 3.2. Example 2: Evaluating Indirect Effects via Bootstrap

**Figure 4:**
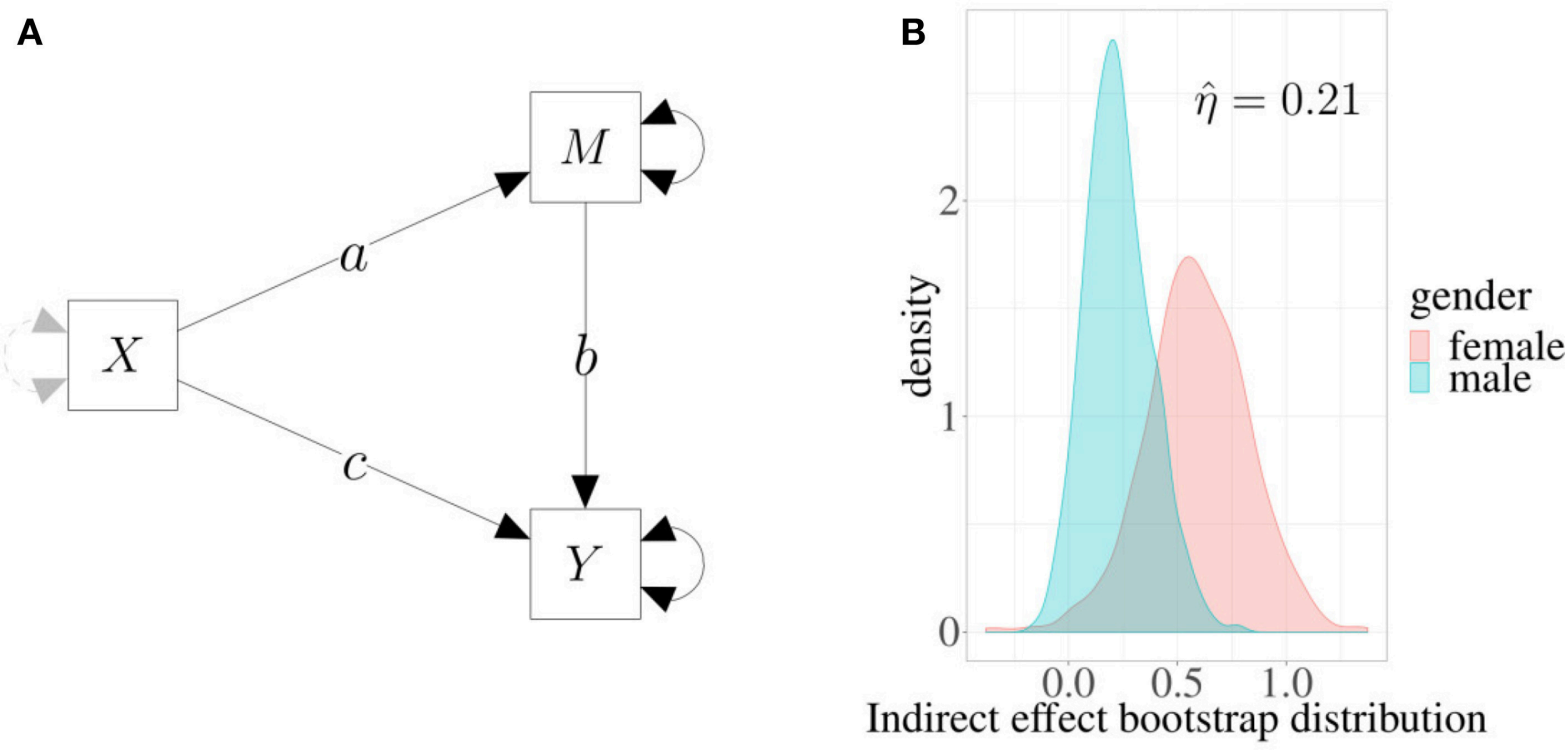
Example 2. **(A)** Mediation model: the effect of *X* on *Y* is mediated by *M*. Single-headed arrows represent regression weights, and double-headed arrows represent variance parameters. The product *a* × *b* is the estimated indirect effect. **(B)** Estimated densities of indirect effects in two different groups based on 1,000 bootstrap replicates.

In Figure 4A, a simple mediation model is depicted in which the effect of predictor *X* on outcome *Y* is mediated by a third variable *M* (see, for example, Preacher and Kelley, 2011). In this representation, single-headed arrows represent regression weights, and double-headed arrows represent variance parameters. Parameter *c* represents the direct effect of *X* on *Y* controlling for *M*, and the product *a* × *b* represents the indirect effect of *X* on *Y*.

We can fit the model on a sample of 100 subjects, 41 males and 59 females. In this case, we are interested in evaluating whether a gender difference exists in the indirect effect of *X* on *Y*. By using a multivariate model, we obtain the following maximum likelihood estimate for the parameters: 0.23 (s.e. 0.15) for males and 0.62 (s.e. 0.26) for females. Now, to estimate the difference between males and females without any kind of distributional assumptions, we can produce the standard bootstrap distributions of the two parameters (for more details, see Rosseel, 2012) and then compute the $\hat{\eta }$ index.

Figure 4B shows the densities of boostrap distributions based on 1,000 replicates for both males and females. The shaded (overlapped) area is about 21%, which indicates that the estimated indirect effect is moderately different in the two groups. Interestingly, in this case overlapping provides a more direct way to assess effects in mediation models, a task which is not always easy to accomplish (e.g., see Wen and Fan, 2015). Moreover, as the index is normalized and is directly computed using the entire distributions of the mediation parameters, its interpretation is simple and straightforward.

### 3.3. Example 3: A Bayesian Analysis

A Bayesian analysis is usually characterized by three steps. First, our degree of belief or uncertainty on parameters is modeled by means of a prior probability distribution. Then, the likelihood of the observed distribution is computed as a function of the parameter values. Finally, the prior information is updated to get the posterior distribution of parameters (e.g., Gelman et al., 2004; Kruschke, 2010; Lee and Wagenmakers, 2013). In the case of well-identified parameters and large sample sizes, reasonable choices of prior distributions will have minor effects on posterior inferences. By contrast, when sample size is small—or in the case where the available data provide only indirect information about the parameters of interest—the prior distribution becomes more important (Gelman, 2002).

In this example, we will consider the Bayesian problem of estimating the mean parameter of a typically symmetric distribution, and we will evaluate how the posterior distribution changes as a function of the prior. In particular, we can consider the typical case of a small sample with 10 observations, with the observed mean being about 0.75. We hypothesize that the true population mean is about 0.5 with two different degrees of uncertainty: (i) a strong prior, Normal(0.5, 0.32), which indicates that we are particularly confident in our hypothesis, and (ii) a weak prior, Normal(0.5, 0.71), which indicates that we are not confident about the hypothesis.

Using Stan (Stan Development Team, 2017), two posterior distributions with 4,000 samples were obtained. The posterior mean estimates were as follows: 0.62, with 90% credibility interval [0.41−0.82], from the strong prior and 0.71, [0.43−0.98], from the weak prior.

By using overlapping index we can now compare priors and posteriors in two different ways. First, considering posterior obtained as an update of the prior, we can evaluate the plausibility of our parameters before and after observing data. Second, by comparing two different priors (strong and weak) and the two associated posteriors separately, we can assess how much the difference computed in terms of priors is the same after having observed the data.

**Figure 5:**
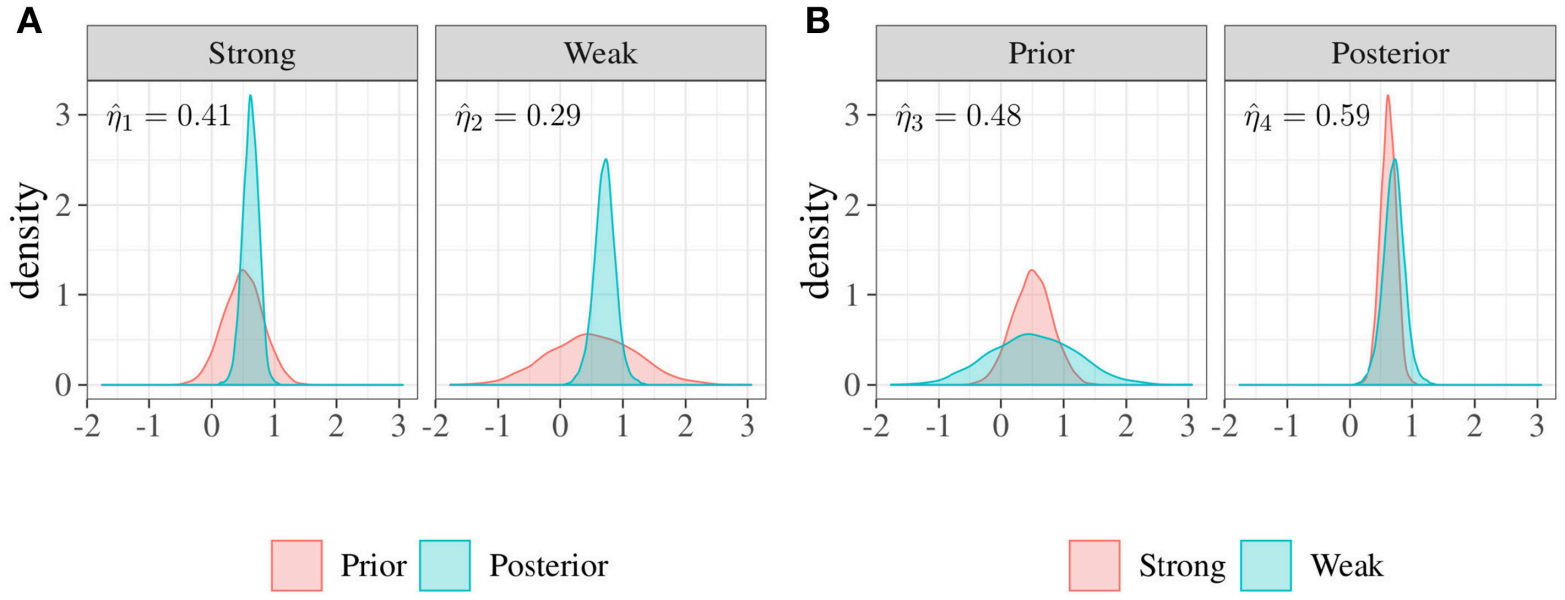
Example 3: Comparisons between distributions of priors and posteriors. **(A)** Priors vs. posteriors. In the left panel the overlap between a strong prior, i.e., smaller variance, and the posterior is about ${\hat{\eta }}_{1}=0.41$, consequently the difference is about 59%. In the right panel the overlap between a weak prior, i.e., larger variance, and the posterior distribution is about ${\hat{\eta }}_{2}=0.29$, consequently the difference is more evident (about 71%). **(B)** Strongs vs. weaks. In the left panel, the overlap between two different priors (strong and weak) is about ${\hat{\eta }}_{3}=0.48$, so the difference is about 52%. In the right panel, the overlap between the associated posteriors is about ${\hat{\eta }}_{4}=0.59$, and the difference is about 41%.

Figure 5A shows the posterior distributions along with the two priors. The left panel depicts the strong prior, i.e., smaller variance, with the related posterior where $\hat{\eta }=0.41$. In this case, the difference between prior and posterior is about (1−0.41) × 100 = 59%. The right panel shows the weak prior with its own posterior. In this case, the index $\hat{\eta }$ is about 0.29 with the consequence that the prior-posterior difference is about (1−0.29) × 100 = 71%. Figure 5B shows the same distributions which are now compared in a different way. In the left panel we can observe the two priors, the overlapping index is about $\hat{\eta }=0.48$, and the difference is 52%. The right panel depicts the two posterior distributions with $\hat{\eta }=0.59$, and the difference is equal to 41%. This result suggests that, even in the presence of a large difference between the two a priori distributions, results do not differ strongly in terms of posterior distributions.

In sum, in the presence of small samples, the overlapping approach can help researchers to assess the impact of their hypotheses in terms of prior distributions on the posterior results. Similarly, overlapping can be used when researchers want to assess posterior distributions of a Bayesian model.

## 4. Conclusion

In this paper, we presented the overlapping index η as a useful measure for quantifying similarities or differences between empirical distributions. This index can be considered as an alternative measure of classical effect size indices, such as Cohen's *d*, Cohen's *U*, or McGraw and Wong's *CL*. In contrast, with these indices η does not assume the normality of distributions nor any other distributional form; in practice, it is usable with any kind of distribution and works properly even in the presence of multimodality. Overlapping can be considered as a similarity measure, defined from the overlapping area (as shown in examples one and two), or alternatively as a difference measure, by considering its complement, 1−η (as shown in the third example).

It should be noted that, since the η index is normalized between zero and one, it can be interpreted similarly to other normalized indices (e.g., correlations coefficients, *R*^2^). In general, η = 0 means that empirical distributions are completely separated; by contrast, η = 1 indicates that empirical distributions are exactly the same. However, for all the other cases, the interpretation depends on the context as suggested by Cohen (1988, p. 25)^1^. Broadly speaking, this index is not intended to be used for inference in a strict sense (i.e., estimating overlapping in the populations); however, it is also possible to obtain uncertainty measures, for instance, using a bootstrap approach.

The η index can be easily computed with the R-package overlapping (Pastore, 2018), which is freely available from the CRAN repository (https://cran.r-project.org/). The package has been recently used in several publications: (1) to evaluate group invariance in the factorial structure of questionnaires in developmental psychology studies using parameter bootstrap distributions (Lionetti et al., 2018b; Marci et al., 2018); (2) to compute a distance index in anthropological measures (Altoè et al., 2018); and (3) to identify group cut-off scores in personality questionnaires, estimating the intersection points of density distributions (Lionetti et al., 2018a; Pluess et al., 2018). Further studies will be needed to analyze the behavior of the overlap measure in situations where regularity conditions are not met. We will postpone this to a future research paper.

## Data Availability

No datasets were generated or analyzed for this study.

## Author Contributions

MP proposed the topic and chose the examples. MP and AC wrote the paper.

### Conflict of Interest Statement

The authors declare that the research was conducted in the absence of any commercial or financial relationships that could be construed as a potential conflict of interest.

## Footnotes

^1^“The terms small, medium, and large are relative, not only to each other, but to the area of behavioral science or even more particularly to the specific content and research method being employed in any given investigation.”
